# On the Margins of Maternity: Low-Income Women’s Experiences of Maternity Care in Late Twentieth-Century Glasgow

**DOI:** 10.1093/shm/hkae011

**Published:** 2024-04-17

**Authors:** Janet Greenlees

**Affiliations:** Centre for the Social History of Health and Healthcare (CSHHH), Department of Social Sciences, Glasgow Caledonian University, Glasgow G4 0BA, UK

**Keywords:** pregnancy, poverty, maternity care, Glasgow

## Abstract

The healthcare provided to expectant mothers impacts the health outcomes of the mother and infant, or infants, and reflects current social and political priorities which mirror middle-class values and leave poorer women feeling socially isolated. Utilising focus group interviews with nineteen women who were living on low-incomes in Glasgow, Scotland, when they delivered their first child between the 1970s and early 2000s, this article analyses the women’s recollections of their maternity care experiences within the changing middle-class health context. It reveals how expectant mothers remembered feeling healthcare practitioners prioritised the needs of the embryo/foetus/infant before their own. The women recalled feeling stigmatised for being pregnant and poor. While interviewees identified individual caring practitioners, overall a disconnect remained between the middle-class healthcare providers and the needs of low-income mothers. Finally, this article suggests that co-creating history with a third-sector organisation could offer a potential methodology for addressing the middle-class bias of official sources.

A pregnant woman’s healthcare experiences influence not only maternal and infant health outcomes, but also her pregnancy journey and individual self-esteem. During the British shift from home births to primarily hospital deliveries by the mid-twentieth century, a middle-class agenda for maternity provision developed. Not only were hospitals staffed by middle-class doctors, political and National Health Service (NHS) maternity initiatives largely favoured middle-class women and continued to do so for the rest of the century. For example, the 1974 NHS reorganisation introduced community-based antenatal care designed to increase women’s choices for and control of their pregnancy care and childbirth experience. This was more feasible for middle-class women who shared the education, values and priorities of policy-makers and practitioners. However, political intent and women’s lived experiences of pregnancy while on low-incomes did not necessarily correspond. This article gives voice to low-income Glasgwegian women’s memories of their maternity care experiences during the late twentieth century. Their stories were gathered through a first-time collaboration between a historian (the author) and Fiona McHardy, the Research and Information Manager for the charity Poverty Alliance, a Scottish network of organisations and individuals working together to combat poverty.^1^ Both policy and historical outputs were planned. However, the key policy findings^2^ need to be understood within the broader context of Scottish and British maternity policy. While the project examined women’s full pregnancy journeys, this article locates their stories of maternity care and social isolation within the broader context of poverty and the British and Scottish political initiatives that sought to address health inequalities and improve maternity care.

Historians have established that maternity care has been largely shaped by middle-class professionals and advocates, which raise questions about marginalised women’s experiences within this framework. Starting with Jane Lewis’ and Lara Marks’ work on early twentieth-century England and later with Angela Davis’ research into women’s experiences of motherhood during the latter half of the century, historians have demonstrated how expectant mothers have been celebrated, but increasingly scrutinised, with middle-class morality at the core of both social policy and healthcare provision.^3^ This scrutiny and the construction of an ‘ideal motherhood’ shifted the balance of power towards middle-class social priorities and practitioners, with a pregnant patient incurring increasingly middle-class expectations concerning her maternal behaviour. This leaves open a vast chasm for disagreement and misunderstanding between middle-class practitioners and their low-income patients, particularly because the latter were largely left out of decision-making about any services introduced on their behalf. As Kiernan et al. and Thane and Evans have demonstrated for unwed mothers, while policy has reflected social perceptions about women’s behaviour, their lived realities differed from the social stereotypes.^4^ During the 1980s, middle-class social policy and medical provision problematised certain social groups, including teenagers, the homeless, ethnic minorities and refugees. As reflected in our participants’ narratives this left many low-income mothers feeling socially isolated.

Sociologist Ann Oakley has argued that the evolution of antenatal care and obstetrics formed part of a strategy for the social control of women.^5^ Oakley’s discussion did not differentiate between women of differing socio-economic status and neither did Marjory Tew’s or Ehrenreich’s and English’s arguments that developments in maternity care emphasised the obstetrical profession’s desire to retain superiority.^6^ Tew also highlighted the importance of the mother’s health during pregnancy towards having a safe delivery.^7^ Here, low-income women and particularly those living in urban areas, faced distinct disadvantages due to their financial inability to secure a healthy diet. While Alison Nuttall and Lindsey Earner-Byrne have demonstrated how poor, pregnant women in Scotland and Ireland, respectively, might have received better food and more rest if they delivered their baby in a hospital rather than at home, these are exceptions within a historiography that has largely ignored poor women’s experiences of maternity care.^8^

Middle-class women’s engagement with maternity services has received scholarly attention. Oakley, Tew, Davis, Mold and others have described English middle-class women’s agency in shaping maternity provision, particularly during the 1980s.^9^ Similarly, Linda Bryder has analysed how women’s groups in New Zealand lobbied the government and hospital management to influence childbirth policies.^10^ The middle-class influence was also evident in antenatal provision. In Scotland, a 1980 Aberdeen study seeking to uncover what comprised routine antenatal care discovered little understanding of its efficacy. Interested parties, including maternity care providers and users, planners and policy-makers, accepted the ‘value of antenatal care’ despite the lack of any clear proof that it can or does achieve its stated intent.^11^ With the middle-classes central to both shaping maternity policy and providing associated healthcare, poorer women were left on the margins of motherhood as defined by healthcare services and policy makers.

Set within this broader landscape of the late twentieth-century British middle-class maternity and policy environment, this article analyses low-income Glasgow women’s experiences of maternity services between c. 1970 and the early 2000s. After first outlining the collaborative research methodology and the Scottish maternity context, the article next analyses low-income women’s experiences of the political initiatives that sought to integrate maternity services. It then turns to the women’s encounters with practitioners and antenatal classes, revealing how while women’s physical needs were met, there was a general neglect of women’s emotional well-being during pregnancy alongside social judgement for being poor, pregnant and sometimes unmarried. Lastly, this article draws some tentative conclusions about low-income women’s experiences of maternity and antenatal care in Glasgow. It suggests that understanding low-income women’s lived experiences of maternity services and the biases within healthcare provision can be helpful for comprehending class-based health disparities, the healthcare experiences of other marginalised groups, and for helping to address gaps in the middle-class bias of official sources.

## Methodology

In 2017 the researchers recruited four focus groups comprising a total of nineteen women who gave birth to their first child in Glasgow between the late 1970s and early 2000s. While Glasgow’s low-income communities are not homogenous, our volunteers were self-selecting and met the project criteria of having been pregnant with their first child while living in an area of Glasgow that ranked within the most deprived ten percent of all Scottish communities.^12^ Eighteen of the women were Caucasian and one was of Pakistani descent. Thirteen women lived with the baby’s father when their first baby was born; four were teenage first-time mothers, and one woman was homeless while pregnant. All participants are referred to by pseudonyms in this article in accordance with the confidentiality measures applied to protect their identity.^13^

The research data was collected through a twofold approach. Prior to each focus group the team explained the project’s aims and requested participants complete a brief survey comprising both open and closed questions about their pregnancy healthcare. The survey offered a reflective tool to help individuals focus for the ensuing group discussion with space for participants to privately share information about their maternity experiences. The information sheets and focus groups enabled us to gather individual pregnancy information alongside broader narrative experiences. Together, these provided the core investigative tools and were chosen because little material about everyday practices, choices and experiences remains in the archival record, particularly for low-income Scots.^14^

We conducted the focus groups in safe, community spaces that were familiar to the women to help them feel comfortable. Indeed, throughout the project the team emphasised creating a safe, non-judgmental space and remained after each session in case anyone sought information about available support and services. Jointly facilitating the group discussions, the team encouraged all women who wished to recount their pregnancy experiences to participate, while also seeking to prevent any dominant voices and minimise the potential for social consensus. The focus groups were designed so that participants could stimulate each other’s memory process, or transactive remembering.^15^ Here, individuals remember certain relationships and interactions but each participant does not remember everything about an event. Memories are formed through the frameworks of social memory.^16^ Furthermore, individual stories are shaped not only by personal experience but also by our families’ collective memories which are transmitted through the stories family members tell, as well as social memory.^17^ While each woman has different memories, collectively their stories may hold similarity. By exploring the same themes with each focus group we could delve deeper than survey research permits to draw out ‘the complexity, the ambiguities, and even the contradictions’^18^ surrounding navigating maternity care systems while living on low incomes.

## The Scottish Context Surrounding Antenatal Care

Scotland’s health and welfare provision has been administratively, although not politically, devolved from Britain since well before the 1947 National Health Service (Scotland) Act and needs to be analysed as such, but within the framework of broader British policy. Many Scots viewed British political healthcare reforms as ‘having no roots in Scottish experience or need’.^19^ Rather than turn to Parliament for answers, many Scottish communities firmly believed that Scots should develop their own methods for tackling health issues.^20^ After devolution in 1997 Scotland’s first First Minister, Donald Dewar, announced that the new Scottish White Paper, *Designed to Care,* would have the objective of ‘patient-centred health care’ for all.^21^ The reformed Scottish NHS would address local needs and circumstances, working in partnership with social work services and housing.^22^ Politicians believed this integrated approach would address multiple issues concerning maternal health and well-being. However, so pervasive was Scotland’s poverty, in 1999 the devolved Government declared a national health priority was to tackle inequalities.

This was not a new challenge. Within Scotland, per capita Glasgow historically has had and currently has the highest concentration of poverty due to the population concentration in the central belt. After Scottish industrial capital collapsed during the 1980s, Glasgow’s unemployment rates remained persistently higher than in other British cities reliant on heavy industry. Indeed, the 1998 Scottish Deprivation Index revealed that Glasgow contained 22 out of the 25 most deprived post codes in Scotland.^23^ Not only have such obstinately high levels of poverty been accompanied by a heavy dependence on government and social services, they have had a detrimental impact on health.

Nowhere is this more evident than during pregnancy. While there were vast improvements in the Scottish Infant Mortality Rate between 1970 and 2010 and which largely followed the pattern of England and Wales (Table 1),^24^ during the 1990s in Scotland, babies born in deprived areas were fifty percent more likely to die and twice as likely to have low birth weights than those born to wealthier households.^25^ Poorer women also tended to have their first pregnancy at a younger age and were more likely to experience stillbirths. Furthermore, low-income communities usually hosted a higher proportion of single and teenage mothers than elsewhere.^26^ In 1985 the Strathclyde region of Scotland had the largest number of one-parent families in the UK with 48,672, most of whom resided in Glasgow. Ninety percent of these lone parents were females^27^ who tended to experience greater financial insecurity than married women.^28^ As evidenced in this article, the social stigma of being an unwed, single mother remained much longer in Scotland than it did in England.^29^ The health, financial and social challenges that many Scottish mothers faced during the late twentieth century helped to shape their pregnancy experiences and associated expectations of healthcare professionals. Yet so too did the women’s poverty shape maternity care providers’ perceptions of their patients.

**Table 1.** Infant mortality rates of Scotland and the United Kingdom per 1,000 live births, 1970–2010

Year	IMR Scotland	IMR United Kingdom
1970	19.6	18.5
1980	12.1	12.1
1990	7.7	7.9
2000	5.7	5.6
2010	3.7	4.2

Sources: Registrar General for Scotland, Annual Report 2000; https://data.oecd.org/healthstat/infant-mortality-rates.htm Accessed 19/05/2022; S. K. Cole and M. Smalls, ‘Trends in Infant Mortality in Scotland, 1970–1987’, *Journal of Public Health Medicine,* 1990, *12*, 1, 73–80, 74.

In an effort to tackle maternal health inequalities the 1977 Labour Government commissioned a Report by the Working Group on Inequalities in Health which was published in 1980. Known as the Black Report after Sir Douglas Black, formerly Chief Scientist at the Department of Health and then President of the Royal College of Physicians, it revealed higher mortality rates among babies of parents born in the lower socio-economic groups across Britain alongside their families’ lower utilisation of health services, particularly those based on prevention.^30^ Indeed, in 1971 only 47.1 percent of Scottish women in the lowest socio-economic bracket had booked any antenatal care by 20 weeks gestation.^31^ During the remainder of the decade, first antenatal bookings gradually occurred earlier in the pregnancy but remained behind those of wealthier women. The Black Report motivated a broader Scottish response to address multiple deprivation. In 1984 the Scottish Health Education Coordinating Committee published recommendations for wide-ranging actions to tackle health inequalities.^32^ Subsequently, various professional bodies, including the Royal College of Physicians and Surgeons of Glasgow (RCPSG), the Health Visitor’s Association and others held related symposia in 1986 and 1987 to galvanise their members.^33^ For example, the RCPSG recommendations mirrored British government reports, suggesting the devolution of healthcare to communities, including maternity care, with community feedback incorporated into planning and operations and resources directed according to need.^34^ In addition, cooperative relations should be developed between the NHS, local authority workers and community groups in deprived areas.^35^ Another report published in 1980, the Short Report from the Social Services Committee of the House of Commons on ‘Perinatal and neonatal mortality’ highlighted how, broadly, the evidence suggested that while working-class people make greater use of GP services for themselves (but not their children) than do middle-class folk, they may receive a lower quality of care. It recognised that antenatal care and the associated uptake required improvements, particularly, but not solely, to address the higher rates of perinatal mortality among both mothers and their foetuses/infants in lower socio-economic groups.^36^ While both reports recognised the need and the potential for changes to healthcare provision, few maternity services’ reforms were initiated.

The Short Report, Black Report and its successor *The Health Divide*^37^ by Margaret Whitehead and published eight years later, all concluded that not only had health inequalities widened since the establishment of the NHS in 1948, but that these were attributable to social inequalities, including housing, education, income, diet and employment. Wide ranging with its recommendations, the Black Report's recommendation number eleven targeted poorer groups’ under-utilisation of community and preventive health services, particularly antenatal care. It suggested that communities should review their services at antenatal and child health clinics and facilities and develop mother-friendly plans to increase their use.^38^ Initiatives should include experimenting with evening and weekend openings and the dispersion of antenatal clinics from hospitals to communities. Lastly, clinics and hospitals should try to humanise both antenatal procedures and the associated environment.^39^ If fulfilled, such reforms may have addressed certain local health needs and priorities.

During the 1980s, a few pioneering maternity initiatives were introduced, including de-centralised antenatal clinics staffed by a consultant and community midwives and workplace antenatal care projects.^40^ In Scotland, the Aberdeen maternity hospital established a routine outpatient antenatal care clinic which proved more popular than the hospital clinic.^41^ In Sighthill, Edinburgh, a scheme shifted antenatal care from the hospital to a community health centre, seeking to tackle the high perinatal mortality rates and low-income women’s high rates of default at antenatal appointments. Women were also allowed a choice of maternity care practitioner—general practitioner (GP), midwife or consultant. Results suggested patient and practitioner satisfaction and a noticeable reduction in local perinatal mortality.^42^ In Glasgow, a hospital antenatal clinic was moved to a peripheral housing estate in an attempt to increase women’s attendance.^43^ However, this quickly became known as a ‘service for deprived people’, stigmatising the women attendees.^44^ Such community maternity initiatives were few and locally driven. Moreover, Margaret Thatcher’s conservative government rejected the entire Black Report because the proposals were deemed too costly.^45^ The political hostility towards tackling inequalities remained with successive conservative governments and was reflected in the attempts to cut welfare provision throughout the 1980s and 1990s.^46^ This responsibilisation or individualisation, alongside the marketisation of healthcare, led to poor people being blamed for their situation. This growing culture of blame and the lack of alternative healthcare initiatives did little towards increasing low-income women’s willingness to engage with middle-class healthcare providers in a healthcare system largely designed to meet middle-class needs and priorities.

Across Britain, maternity care became premised on the idea that antenatal care was beneficial, but that delivery could be refined. The Scottish government restructured primary care to offer more comprehensive services delivered by extended community teams to include antenatal, postnatal and child health services.^47^ Initiatives included introducing ‘commando groups’ to round up high-risk mothers for antenatal checks through compulsion or bribery.^48^ While there may be some crude logic to this strategy, not only is it morally and ethically dubious, it did little towards improving patient-practitioner relations or ensuring high standards of care. Indeed, the Short Report had confirmed that such a strategy ignored the primary contributors behind perinatal mortality, namely economic deprivation, high parity and extremes of age—all of which were most common among low-income women.^49^

In 1998, the Blair Labour Government’s *Report of the Independent Inquiry into Inequalities in Health* (chaired by Sir David Acheson) also confirmed that antenatal care and its uptake required improvements and targeted the higher rates of perinatal mortality among mothers and their foetuses/infants in lower socio-economic groups.^50^ The Acheson Report influenced the 1998 Scottish Green Paper *Our Healthier Nation: A Contract for Health* which aimed to reduce health inequalities. However, during the 2000s, public health policy emphasised changing lifestyle behaviours not healthcare provision. Indeed, while both British and Scottish Health Policy repeatedly recognised maternal health inequalities and promoted service integration, as this article highlights, women living on low-incomes were frequently unaware of maternity policies. Moreover, their implementation did little to improve low-income women’s experiences of maternity care provision.

## Integrated Maternity Care and Women’s Memories of Maternity Choices

Integrated maternity services were designed to increase patient choice and to provide community healthcare and local cooperation between healthcare professions. Scottish efforts at integrated maternity services did not address the underlying issue of middle-class inter-professional rivalries between GPs, midwives and obstetricians. Indeed, each profession believed that ‘*it* was the best one to offer women information, advice and reassurance’.^51^ The struggles for professional authority and autonomy resulted in fewer regions managing to offer integrated maternity care and created patient confusion about providers and provision. This, in turn, inhibited patient choice and was particularly evident in low-income communities where residents relied on local healthcare services due to travel costs, time constraints and familiarity. Our focus groups memories of maternity care provide a Scottish example of Alex Mold’s argument about the limited successes of political efforts to increase consumer choice across Britain.^52^ Instead, certain structural challenges served to disempower the Glasgwegian expectant mother including poverty, factors of place, including social expectations of maternity behaviours, and ongoing professional rivalries, although the latter is not a focus of this article.

In 1974, the newly formed Greater Glasgow Health Board discontinued local authority community antenatal clinics. From this point, general practitioners and hospital-based obstetric specialists introduced ‘parallel care’ resulting in considerable duplication of effort. By 1980, hospital-based antenatal clinics had become renowned for their ‘long waiting times, difficult access to clinics, lack of continuity of care, [and the] lack of opportunity to discuss things that women themselves are worried about’. Women did not feel that they were getting as much from their antenatal appointments as they thought they should.^53^ Parallel care had shifted the pregnancy experience from a familiar community environment to a middle-class hospital setting, thereby enhancing existing class disparities between low-income patients and middle-class practitioners. This new system resulted in missed appointments with women feeling that they had a robotic experience of maternity care, similar to the women in Hall, Macintyre and Porter’s Aberdeen study.^54^

Despite political attempts to improve antenatal care and increase patient choice, few of our interviewees remembered having choices for their maternity care. Antenatal appointments at either their doctor’s surgery or the hospital were with ‘whoever’s available’.^55^ Most women utilsed the hospital antenatal clinics and remembered seeing multiple healthcare professionals when pregnant. Usually, a different midwife attended each antenatal appointment. Sylvia, who delivered the first of three children in 1988, remembered how for all three pregnancies: ‘You never had a choice [of care provider], that was kind o’, you just did’. Michelle, who also gave birth to her first child in 1988, agreed, ‘I’ve no’ heard anybody recently had anything [choice]…’. For routine appointments, ‘They had no choice… It was almost robotic, it was like this is the process…’.^56^ Participants also remembered having multiple health visitors after their baby was born.^57^ While continuity of care was not necessarily standard health board practice, the lack of it hindered relationship formation and hence the willingness of patients to ask questions or to request care preferences.

Although the lack of choice of practitioner was not a class-based experience in a cash-strapped, over-stretched maternity service, expectant mothers living on low-incomes faced the additional strains of travel costs, potential childcare costs and time to attend the hospital clinics, resulting in missed appointments.^58^ Indeed, three other participants who were pregnant during the 1980s remembered having no antenatal checks, although two claimed that they did not realise they were pregnant until very late in their pregnancies. However, the women contended that they would have struggled to attend appointments due to lack of time and money.^59^ While four participants did not remember attending more than two antenatal appointments, four others could not remember how many antenatal appointments they attended and six recalled attending more than five.^60^ Two women explained that they missed some appointments due to the lack of childcare and work commitments.^61^ Across the decades the continuity of experiences was clear. Participants remembered wanting to engage with maternity care, but circumstances including time, travel costs, wages sacrificed and childcare meant they sometimes felt unable to do so.

As patient consumerism became increasingly central within Scottish health policy during the 1980s and 1990s, the rights of the individual increasingly superseded the rights of communities.^62^ Yet our participants did not consider themselves to be healthcare consumers with the ability to shape their own pregnancy experiences. While they intimated that increased maternity care options would have been preferable to simply seeing ‘whoever’s available’,^63^ at the same time, participant’s experiences of healthcare did not diverge from expectations. They were not used to having healthcare choices and hence, did not expect anything different when pregnant. Nevertheless, when the higher rates of maternal and infant mortality and pregnancy complications in low-income neighbourhoods are combined with the number of women in these communities who attended few or no antenatal appointments, it is clear that broader community maternity needs were not being met.

Similarly, few women remembered having choices about where they gave birth. After delivering her first child at Glasgow’s Rottenrow Hospital in 1987, Jane refused to consider anywhere else for birthing her later children. In 1995, Siobhan was offered the choice of delivering her second baby at either the Rottenrow or the Queen Mother’s Hospital. She chose the latter because that was where she had delivered her previous baby. It was also where she had been born. Yet the majority of women, like Naomi, remembered how ‘We never got a choice…. I just went to the nearest one [hospital]’. Asali remembered how in 2006 she also ‘just took the nearest one’.^64^ Consistent across the three decades, women believed that the lack of choice for birth location was simply the current state of maternity provision in the city. While the few participants who were offered a choice of hospital seemed appreciative, the interviewees’ broader ambivalence about the birth site should not suggest a lack of desire for choice. Rather, women remembered how their birth priorities centred around the event, not the location.

Maternal choice surrounding the birth experience was also limited. At least six women clearly recalled not being asked whether they preferred a midwife-led or a doctor-led birth and many remembered not having their desired birth experience. Alice, who delivered her first child in 1996, recalled, ‘I never got any choice, it was just a case o’ [who’s] there, it, well, noo it’s midwife led’.^65^ In 2004, Kirsty had requested a water-birth on her birth plan. When she arrived at the hospital in labour she was told ‘oh, too busy, you’ll no be gettin it…’.^66^ Several other women were adamant that they lacked choice surrounding childbirth and spoke in aggrieved tones. It is unclear whether birth plans were a 1980s apparition reflective of the drive to increase patient choice or whether an over-stretched maternity service made birth plans an illusion. Nevertheless, patients sought practitioner respect for their wishes, even when practicalities prevented implementation. The lack of choice during the pregnancy journey does not necessarily reflect a class-based experience, it simply could be symptomatic of an under-funded and over-stretched maternity service. Indeed, Angela Davis found that pregnancy care choices were not guaranteed and the use of assistive birth technologies was common among her largely educated and middle-class English mothers.^67^ Rather, poverty exacerbated the Glasgow woman’s experiences in the middle-class medical environment through social stigma and practitioner assumptions about maternity and motherhood. While historiography has emphasised how hospital policies and practices determined birth experiences and the use of assistive technologies rather than medical need,^68^ less visible are the socio-political expectations surrounding maternity. Yet these hold considerable potential for marginalising women based on income, race, disability and marital status.

Hospital policies and circumstances outside the women’s control were particularly influential in shaping the birth experience. For example, hospital policy prevented a woman from having a water-birth if she had been induced.^69^ Other times circumstance limited patient choice, including when Kirsty had arrived to a full labour ward. While the care offered individual women during labour may have followed hospital policy, a pregnant woman may not understand that the use of certain assistive technologies during childbirth was optional or that medical intervention might serve practitioners’ interests more than the patients.^70^ British women were supposed to have input into what medical technologies, if any, doctors and midwives used during labour.^71^ Jacqueline Wolf has argued how because certain technologies offered both time and cost efficiencies to healthcare workers, they were sold to hospitals in the United States as a way to reduce the total number of attendances. In some cases, their use became standard practice.^72^ When our focus group participants discussed the relationship between hospital policies and maternal choice, medical technologies and interventions were not mentioned. This may suggest different priorities to Davis’ English interviewees who recalled the importance of choice during pregnancy and birth in the 1970s and 1980s, particularly with relation to the use of medical technologies. Her participants expressed dissatisfaction with the lack of information about the different technologies, the way in which they were employed, and the attitude of some hospital staff.^73^ In contrast, many of our interviewees who experienced medical interventions during pregnancy and childbirth remembered accepting these as necessary for theirs and their baby’s well-being.

Our participants memories centred on the people they encountered during antenatal appointments, labour and immediately postpartum. While the women believed healthcare choices were important, encounters, relationships and rapport with practitioners were more significant. While Davis’ interviewees also recalled practitioner encounters and were frequently very critical of their treatment by ‘self-important health professionals who believed that they, rather than their patients, knew best’, these largely related to experiences during labour and the immediate postnatal period.^74^ Nevertheless, both Davis’ and our interviewees believed the communication between patient and practitioner was central for a woman to understand her pregnancy and any suggested procedures. While communication is not a class-based concern, class differences influenced relationships. At antenatal appointments Davis’ interviewees were more willing to ask questions of the middle-class practitioners than our participants.^75^ Our interviewees remembered not wanting to impede on a practitioner’s time. Women also recognised the social and educational gaps between themselves and the practitioner and feared the responses to questions. Two participants vocalised how they thought ‘the nurses and doctors knew best’. The rest of the focus group agreed with comments including: ‘Aha, that’s true’, ‘simple as that’, ‘Mm Hmm’, ‘What do I know, I mean…’ and ‘I’d never had a baby before’.^76^ Another group offered similar responses to the question ‘did you feel that you were able to ask any questions you wanted with the midwife and GP?’, including: ‘No, no I didn’t’, ‘No, it was definitely, you were embarrassed, you know what I mean’, ‘you were feared to ask them anything when you went’.^77^ The women feared being judged as ignorant by the doctor or midwife and lacked the confidence to seek the advice they wanted, particularly when they were unable to form a relationship with a regular antenatal care provider. Into the 2000s, class-related communication struggles remained within maternity provision.

## Low-Income Women’s Recollections of Maternity Care

As Hall, Macintyre and Porter have acknowledged, studying patient ‘satisfaction’ with healthcare, and particularly any one type of healthcare such as antenatal, contains conceptual and methodological problems—not least because individuals have different definitions of satisfaction.^78^ Rather than explore interviewees’ satisfaction with their maternity care encounters, we sought to understand women’s experiences with pregnancy care providers. Across the focus groups participants sought reassurance that the practitioner was interested in the mother’s physical and emotional well-being separate from that of the embryo/foetus. Yet desiring personalised and attentive maternity care is not unique to Glasgow. Communicating kindness and concern affirms the inherent dignity of all expectant mothers.^79^

Physical and emotional well-being are intimately connected, particularly during pregnancy. The emotions surrounding the biological changes and impending motherhood suggest that antenatal care should address both.^80^ Such provision is dependent on the efforts and attitudes of individual practitioners. While this is true for all women, low-income women are particularly vulnerable because of their greater health and social welfare needs and because they are reliant on available local provision. Our participants’ memories of their clinical encounters varied with poverty proving central and encounters frequently at the extremes. Some medical professionals and welfare providers neglected the woman’s emotional well-being and focused purely on the physical health of the unborn foetus. Others went above and beyond their duty in caring for the expectant mother.

During the late 1980s, four participants turned to a Catholic project in Glasgow called The Innocents for maternity support, but with different experiences. Founded shortly after the 1967 Abortion Act by a group of private citizens concerned about perceived societal trends towards automatic abortion on request, the Innocents, a pro-life group, sought to offer mothers-to-be practical help to keep their babies. While initially Catholic, by 1980 the Innocents was an interdenominational organisation. The Glasgow branch was run by the Franciscan Sisters Minores, based at the Saint Francis Maternity Home in Govan.^81^ Here, the nuns provided both hospital maternity care and welfare support. Three interviewees, all pregnant out of wedlock in 1987 or 1988, found the Innocents very helpful. They received healthcare, welfare support and the space to discuss their options.^82^ These women also had supportive families and which may have influenced the nuns’ approach.

Michelle had a different experience with the Innocents. She had been brought up in care and was nineteen and homeless when she became pregnant in 1988. She turned to the Innocents for help because years earlier they had supported her pregnant mother. Michelle sought to use their temporary accommodation available for homeless and pregnant women. While the charity provided Michelle with some practical help and support, she recalled feeling that the staff prioritised the baby’s well-being before hers.^83^ Moreover, the nuns encouraged Michelle to put her baby up for adoption, possibly because she was homeless. Wanting to keep her baby Michelle left the facility, choosing to either sleep rough or to ‘sofa surf’. She believed that the combined stress of homelessness, pregnancy and the push to have her baby adopted contributed to her developing bulimia and pre-eclampsia. Michelle credited the Glasgow midwives for helping her.

So the midwives, the midwives chasin’ me all over Glasgow, you know, tryin’ to find me and, you know, ‘where are you?’, ‘where is she?’, and y’ know, ‘you really need to be getting…’… ‘they were really interested in my health, it was in me, you know, I felt as if it was nothin’ to do wi’ the, the child, it was me. They were probably more interested in where I was and, you know, where I was located, eh, but as I say, I left the Innocents because o’, eh, their treatment, em, they were basically tryin to get a family lined up and everything.^84^

These midwives were not unique with their extensive efforts to ensure a vulnerable woman received antenatal care. During the 1970s, Jan Fenton, a community midwife in Whitfield, a low-income neighbourhood in Dundee, would appear on the doorstep of women who defaulted on their antenatal appointments. She recalled one encounter:

We missed you at the clinic today. Are you OK? One day I heard this chap [in the clinic] saying, ‘Oh we thought we’d better bring her to the clinic or that wumman wid ha bin at the door again’. But then you see they began to realise that there was somebody who was thinking that it was important and they needed the care.^85^

These midwives’ efforts were all the more remarkable when couched within the context of a continued over-stretched and under-funded Scottish maternity service where the boundaries of midwives’ professional authority and autonomy were unclear. When a mother entered the hospital environment she relinquished her authority, whereas in the home the mother retained the status of host that student midwives had been taught to respect.^86^

All interviewees believed that in order for practitioners to secure a woman’s engagement with maternity care, they needed to treat each patient as an individual. Michelle not only needed antenatal care but she found that knowing someone cared about her as a person and not merely as a vessel for the baby was important to her well-being as both a woman and as an expectant mother. Michelle recalled how the midwives ‘were the only wans that looked oot for somebody like me that was homeless’.^87^ Other participants who delivered their first baby during the 1980s through early 2000s also remembered how one individual healthcare professional could make them feel valued, particularly when in vulnerable circumstances. This could comprise a midwife or health visitor making more frequent postnatal visits to ensure the new mother was coping or to provide additional help with breast or bottle-feeding.^88^ While caring for an expectant woman’s health and mental well-being is not class related, many low-income women had few support networks and limited financial resources. When combined with wealth-related health disparities, the clinical encounters became particularly important to the women.

All focus groups discussed how attentive maternity care proved critical for minimising their social isolation within the healthcare system and for providing emotional and practical support.^89^ Participants also remembered how it was exceptional to meet practitioners who valued the expectant mother as an individual, as Michelle had. One practitioner had the ability to build or destroy an expectant mother’s confidence. While any expectant mother could experience a poor clinical encounter,^90^ for women living on the margins, negative patient-practitioner encounters could reinforce the stigma of poverty. For example, after finally securing a council flat at eight months pregnant, Michelle was feeling much more positive about her health and the future for herself and her baby. She regularly attended specialist antenatal clinics at the Easterhouse Health Centre to address her pre-eclampsia. Michelle remembered at one appointment overhearing the consultant telling his nurse that ‘before he retires, he’s gonnae get every single young person in Easterhoos he called it, sterilised, every single,… so I felt as if I were, were, were nothing’.^91^ Yet the consultant knew Michelle’s baby was much wanted. Despite his comments raising her emotional anxieties, Michelle did not criticise her medical care. Rather, it was the practitioner’s lack of emotional sensitivity and judgment that made her feel isolated. The other participants in Michelle’s focus group also recalled having felt humiliated by certain charities and doctors they encountered during pregnancy, simply for being poor. Michelle had the additional stigma of being young, single and recently homeless.

The medical profession's lack of sensitivity towards difficult pregnancies was another common thread across the focus groups. Siobhan’s female relatives had a history of problematic pregnancies and doctors had informed her that she was unable to have children. When in 1987 Siobhan discovered that she was pregnant and visited her GP, he told her ‘don’t bank on keeping this baby’ and advised her to consider an abortion. Siobhan’s was a much-wanted pregnancy so the doctor’s attitude caused her significant emotional stress. She recalled, ‘it was the way, way the doctor had worded it tae me, you know, like…’. His attitude also increased Siobhan’s fears that she might miscarry. Siobhan felt judged by her GP and added, ‘em…, I was goin’ on twenty-one, I wasnae a silly wee lassie…’.^92^ While it was unclear whether the doctor was merely concerned about Siobhan’s health if she continued the pregnancy, Siobhan’s feelings were similar to those of other participants. Namely, many practitioners disregarded how pregnancy was an important time in the women’s lives. They failed to view the women as mothers. Whether accurate or not, participants recalled feeling that many middle-class practitioners looked down on the women for being poor and seemed to correlate poverty, youth and unwed motherhood with being a bad mother.^93^ By implication then, marital status, age and income helped to define what comprised a good mother.

The stigma of being a bad mother due to poverty contained various elements, including using charity shops to meet their maternity needs. Women in all focus groups relied on charity shops to prepare for motherhood and which made them feel embarrassed.^94^ Michelle recalled ‘I was embarrassed for people to know that [I was poor], ‘cause I, I didnae have a pram either, you know, em [background agreement]’. However, Sylvia was more pragmatic remembering that depending on charities ‘It was the situation’.^95^ Either way, the shadow of poverty crept into all facets of the women’s pregnancy experiences and motherhood, including their mental health.

Participants across all focus groups recalled how an individual practitioner could make them feel a failure as a mother. Sara remembered how after her son was born in 1990 the health visitor made her feel a failure as a mother simply by expressing concern about her baby.

[I]t was frowned upon as well, you know, you were a mother now, so it was sort of frowned upon if you were comin’ up wi wee things like, aw, like, well, he’s been greetin’, at night and, you know, it was seen as if you were just, like, you were a failure, you were a moan… I think it was kin o’ frowned upon as well if you werenae coping, you now…(murmurs of agreement from other group participants) as, you know, ‘cause you, it was seen as if you coped, and that’s it, you got on wi’ it, you know, it’s like, ‘you’ve made your bed and you lie’, you know.^96^

At this time, Sara had a strained relationship with her partner, so the health visitor’s words made her feel even more alone. Indeed, women in all focus groups remembered how when a new mother, if a midwife or health visitor passed judgement about anything, no matter how trivial, it made them feel isolated and that they just had to ‘get on wi’ it’.^97^ While Angela Davis’ English interviewees were also critical of their health visitors, this was because they felt neglected, not judged.^98^ Whether class played a role in attitudes is both unclear and individual. However, our participants found the health visitor’s judgement particularly difficult because they were poor, frequently unwed and/or without family or partner support. They did not meet the Scottish social expectations of motherhood.

Individual personalities and an over-stretched health and welfare system may have prevented practitioners having the time to provide women with additional support. Nevertheless, participants clearly remembered feeling stigmatised as ‘bad mothers’. One woman remembered ‘then you think, and then it’s a vicious circle, ‘cause you’re a bad mother, you’re no’ copin’, [you think that] everybody else is copin’.^99^ Betty, who was a single mother aged nineteen when her first baby was born in 1985, remembered how both the midwife and health visitor made her feel ‘I cannae do this, I’m a bad, I’m a bad mother’, simply because they ‘was going’ up to the hoose every week, you know?’^100^ At the time, Betty thought the regular visits from the midwife and health visitor related to her being a ‘bad mother’. On reflection, Betty realised that the frequent visits stemmed from professional concern about her postpartum mental health because the doctors had put her on these ‘type o’ tablets’ because of ‘the way she was feelin’.^101^ While not all participants discussed their maternal mental health, those that did had been particularly vulnerable during pregnancy, being young, single, poor, sometimes in a troubled relationship and/or lacking family support. During the late twentieth century, maternal mental health and particularly postnatal depression, were the silent maladies of maternity. This may have enhanced the women’s memories about unmet emotional needs as they struggled to navigate the effects of deprivation in a society with rigid moral expectations about motherhood.

Our participants memories about the moral overtones of individual practitioners and charities were reflective of wider Scottish society.^102^ As Roger Davidson and Gayle Davis and other historians have demonstrated, both Scottish politics and Scottish society retained a strong, clear, moral overtone towards maternity. It also remained for much longer than in England.^103^ The 2000 Scottish Social Attitudes Survey revealed that the majority of respondents not only considered marriage to be the best kind of relationship, but that people wanting children should marry. Furthermore, most people believed that the levels of teenage pregnancy in Scotland were problematic.^104^ Many Scots continued to frown on abortion decades after the 1967 Abortion Act, believing that the legislation failed to meet the needs or values of Scottish society.^105^ Into the twenty-first century, and despite dwindling church congregations, the moral agendas of the Scottish Churches, including the Presbyterian and Roman Catholic hierarchy, continued to exert significant influence on both national and local legislative processes, as well as throughout society.^106^ In this social context it is unsurprising that many of our participants felt particularly vulnerable.

The provision of antenatal care in late twentieth-century Scotland emphasised a woman’s physical care.^107^ Holistic care, to include awareness of the expectant mother’s emotional needs, was lacking. However, our interviewees were unsure whether changing attitudes towards mental health during the twenty-first century influenced their belief that maternity care must combine physical and emotional elements, including pregnancy anxieties, the ‘baby blues’ and postnatal depression. Nevertheless, when discussing their own mental health, participants remembered how ‘it was just not discussed’.… ‘yeah’.… ‘definitely’.^108^

Social and moral expectations surrounding women’s behaviour can overshadow and leave unmet their physical and emotional needs, not just concerning unwed mothers, or the postnatal period, but also during difficult pregnancies. For example, in 1984 doctors realised that at 28 weeks, Jenn’s baby was dying in the womb. Yet, they insisted that she carry the baby to full term. Jenn remembered how the doctors offered no rationale, emotional support or counselling at appointments, when her baby was stillborn, or after the hospital buried her baby and refused to let her erect a headstone.^109^ During her journey to come to terms with her loss Jenn remembered meeting other women who had undergone similar stillbirth experiences. Jenn’s encounters with the medical profession’s lack of emotional sensitivity recurred in a later pregnancy when she learned that her foetus had Down’s Syndrome. Jenn remembered how ‘[t]hey [medical staff] wanted me to get the baby aborted’. She refused. Again, Jenn was not offered counselling or emotional support for the decision-making or to adjust to her new daughter.^110^ Moreover, she felt that the medical staff displayed a ‘doctor knows best’ attitude. Both Jenn and Michelle agreed that the medical staff ‘always detach their selves fae that emotional side o’ things, do you know what I mean? It’s like there, there, it’s a gap, there’s, there’s just naebody that picks it up’.^111^ Indeed, Scottish maternity care reflected late twentieth-century middle-class social expectations surrounding motherhood and was couched within the wider social silence surrounding emotions, mental health and pregnancy.

Practitioners’ perceived lack of compassion emerged in other situations. Women who had had miscarriages remembered the term ‘product’ being used to describe the miscarried foetus, yet the mother considered this a child lost.^112^ Other women recalled that after a stillbirth or if their baby had to go to the hospital’s special care unit, they were returned to the general maternity ward with other mothers and their healthy newborns.^113^ While these are not class-based experiences, the lack of practitioner empathy for maternal feelings of loss or separation were exacerbated for marginalised women with few additional support networks and no funds to pay for professional counselling. When reflecting upon their experiences, interviewees emphasised the personal impact from both poor communication and interpersonal relationships before any medical procedures. Positive patient-practitioner relations could have helped the women to navigate the effects of deprivation and limit social isolation.

Improving patient-practitioner relations requires empathy and regular encounters between the two individuals, something our interviewees rarely experienced. While health policy did not require continuity of care provider, the lack of familiarity or an established patient-practitioner relationship left individuals ‘feared to ask them anything when you went’.^114^ Instead, memories about the impersonal medical culture surrounding pregnancy dominated. Participants recalled hospital maternity units where midwives operated ‘like [the women were on] a conveyor belt’,^115^ something Lindsay Reid described as being a product of the shift to hospital births during the 1930s through 1950s.^116^ Yet it was not only expectant mothers who felt practitioners believed the process of pregnancy and birth was routine and generic rather than a special event for each woman. Reid’s study of twentieth-century Scottish midwives found that many midwives felt the same. They believed the caring that had been integral to midwifery for centuries was disappearing with the move to hospital births. A midwife who worked during the 1980s and 1990s recalled:

I always feel that women, when they are pregnant, when they are in labour and just after, need to be mothered themselves in order to help them to mother, even if they have got a mother-figure in their own family. They need the caring that goes alone with midwifery. There was no caring. The women were delivered—it was just like a sausage factory.^117^

Policy was dictating maternity care.^118^ Yet the policies implemented did not address the maternal health inequalities identified in the Black Report, the Health Divide, the Acheson Report or the 1998 Green Paper. By 2000, ninety-nine percent of Scottish births occurred in a hospital.^119^ The shift to hospital centred maternity care not only blurred the role of the midwife in childbirth, it weakened the patient-practitioner relationship due to lack of community familiarity.^120^ This, in turn, contributed to some patients experiencing social isolation with tensions arising between the expectant mother and the attending practitioner.^121^ More broadly, the poor patient-practitioner relations contributed to maternity care and caring becoming generic rather than meeting the needs of individuals.

## The Inadequacies of Antenatal Classes in Meeting Maternal Needs

Maternity care does not simply encompass encounters with healthcare professionals for antenatal checks, childbirth and postnatal care, it also comprises health education. Scottish social and moral expectations of motherhood were also reflected in antenatal classes. Along with their antenatal appointments, the classes further challenged our participant’s emotional strength for navigating both motherhood and deprivation. While three respondents remembered how antenatal classes provided helpful preparation for childbirth and motherhood, most felt out of place in these classes because of their poverty and/or because they were unwed mothers. Attending antenatal groups was largely a middle-class expectation of appropriate maternal behaviour which encompassed middle-class goals and values concerning family and motherhood.^122^ Our participants could not afford nice maternity clothes or new baby equipment and felt visibly different and unable to participate in antenatal classes with wealthier mothers.^123^ When Fiona first attended antenatal classes in 1990 she found ‘it was a’ the snobs’. She decided ‘I’m no’ goin’ back to that, ‘cause they looked doon their nose at you, sittin’ there with their husbands and you were sittin’ there on your tod’. Liz and Alice agreed that the atmosphere in the classes was ‘snooty’. Liz remembered how she ‘Just felt stupid, you know…. I went to one with my first and I was like, I’m not comin’ back to that’.^124^ Yet antenatal classes were intended to welcome expectant mothers into a new social network of motherhood, something promoted by the National Childbirth Trust (NCT). While the NCT was a national organiser and promoter of antenatal classes, it was also run by middle-class women^125^ and did not meet the needs of marginalised women.

Because antenatal classes ignored the lived realities of women struggling to balance pregnancy with navigating the challenges of poverty, our participants were left feeling further marginalised from the broader network of motherhood. This helped to reaffirm the ongoing Scottish moral assumptions and social anxieties surrounding marriage and maternity, whereby marriage and financial independence were the social circumstances in which a woman should become a mother. Pat Thane and Tanya Evans found similar beliefs in England concerning unmarried motherhood. Single mothers, particularly teenage mothers, were viewed as the ‘archetypical welfare scroungers’.^126^ During the 1980s and 1990s when most of our interviewees had their first child, many Scots—including some politicians—believed the welfare system was encouraging marital break-up and lone parenthood. While individual Scots were becoming increasingly tolerant of unconventional families, public and collective social and political condemnation of them remained.^127^ This left many women, including our participants, feeling socially isolated.

## Conclusions

The Black Report’s call for the ‘humanisation of antenatal procedures and settings’ failed to take hold in late twentieth-century Glasgow.^128^ Engrained within Scottish maternity care and associated policy were a century of social expectations surrounding pregnancy and motherhood. Our focus group participants highlighted the practical and emotional struggles they faced during pregnancy and the failings of the healthcare system when they did not meet social expectations. These experiences may have found resonance with other women’s experiences of maternity care in impoverished or otherwise marginalised circumstances in Glasgow and beyond. Of our participants, the young and single mothers felt particularly stigmatised and judged by healthcare professionals and society as they tried to navigate the complexities of low-income maternity and motherhood. Participants experiences were also characterised by contradictions, particularly as some encountered individual practitioners that went ‘above and beyond their duty’ to help them when vulnerable.

While poverty overshadowed participants experience of pregnancy, the women did not feel that poverty overshadowed their motherhood. They spoke proudly about their children who had grown, or who were growing, into confident young people. For example, during one post interview chat Mary proudly told us that her son, born out of wedlock when she was aged sixteen, was soon to graduate from University.^129^ Participants also readily laughed about the challenges they had faced while pregnant and as young mothers, recognising how far they had travelled on their journey. Yet the experiences of marginalised mothers remains hidden in history and health and social policy. The extensive historiography surrounding maternity and motherhood has largely overlooked any associated experiences of deprivation. This article helps to address this omission by uncovering some of the nuances of low-income women’s relationship with medicine where existing health and policy frameworks were staffed by highly educated professionals with little knowledge of the lived realities of life on low income.

Broader health policy also requires nuance for the Glasgow context. Late twentieth-century healthcare initiatives were short-lived and their histories ignored,^130^ including those in Aberdeen, Sighthill and Glasgow. By the 2000s, the policy landscape surrounding maternity and low-income had scarcely changed. The morally charged issues of teenage pregnancy and sexually transmitted diseases continued to stigmatise Scottish women on low-incomes^131^ and impact their experience of healthcare. Social isolation and the role of wealth in reinforcing social expectations of maternal behaviours have been largely overlooked by the growing number of studies which have uncovered many of the nuances concerning the relationships between women and medicine.^132^ Future research needs further investigations of marginalised women’s experiences of healthcare and to move away from the current focus on health policy and its implementation.

Furthermore, the centrality of poverty to our focus group participants lived-experiences of maternity between the 1970s and 2000s suggests the potential benefits of our methodology as a way to help fill gaps in the historiography and influence policy. Co-creating research and understandings of historical experiences with a third-sector organisation suggests that the integrative approach adopted for this article could help to develop much needed additional histories of the lived experiences of people on the margins of society, whether from pregnancy, disability, mental health, race, poverty or any combination of issues. This project elicited a policy brief which reached government officials, third-sector organisations working with marginalised communities and the Deep End GP network—a network of General Practitioners who serve Scotland’s 100 most deprived communities, as defined by the Scottish Index of Multiple Deprivation (SIMD).

Future collaborations between historians and the third sector could help to uncover the extent to which policy has addressed the needs of those it intends to help. Here, this method highlighted how maternity services do not currently meet the needs of women living on low-incomes. This is not to imply inequalities in the physical care provided, but rather that service location, patient-practitioner interactions and information about pregnancy services, choices and welfare need reforming to more directly meet the needs of all women. Recollections of lived experiences of maternity on low-income should inform the development of new policy guidelines for maternity policies that do not simply mirror middle-class values and problematise individuals. Indeed, the co-creation of research between academics and the third sector holds broader potential for influencing health and social policy and reducing social isolation and health inequalities.

## Footnotes

1
https://www.povertyalliance.org/. Accessed 23 May, 2022.

2 Fiona McHardy with Janet Greenlees, *For Mothers and Babies: Pregnancy and Antenatal Care – Low income women’s experiences during the 80’s, 90’s and early 2000’s (2018) https://www.povertyalliance.org/wp-content/uploads/2019/03/Failing-Babies-Poverty-maternity-and-healthcare-in-the-twentieth-century_fin..pdf. Accessed 23 May, 2022.*

3 Jane Lewis, *The Politics of Motherhood: Child and Maternal Welfare in England 1900–1939* (London: Croom Helm, 1980); Lara Marks, *Metropolitan Maternity: Maternal and Infant Welfare Services in Early Twentieth Century London* (Amsterdam: Rodopi, 1996); Angela Davis, *Modern Motherhood: Women and Family in England, 1945–2000* (Manchester: Manchester University Press, 2012).

4 K. Kiernan, H. Land and J. Lewis, *Lone Motherhood in Twentieth-Century Britain* (Oxford: Oxford University Press, 1998); Pat Thane and Tanya Evans, *Sinners? Scroungers? Saints?: Unmarried Motherhood in Twentieth-Century England* (Oxford: Oxford University Press, 2012).

5 A. Oakley, *The Captured Womb: A History of the Medical Care of Pregnant Women* (Oxford: Basil Blackwell, 1984), 2, 250.

6 Marjory Tew, *Safer Childbirth?: A Critical History of Maternity Care* (London: Chapman and Hall, 1990); Barbara Ehrenreich and Dierdre English, *For Her Own Good: 150 Years of Experts’ Advice to Women* (New York: Doubleday, 1979).

7 Tew, *Safer Childbirth*, 199–201, 209–10.

8 Alison Nuttall, ‘Taking “Advantage of the facilities and comforts… offered”: Women’s Choice of Hospital Delivery in Interwar Edinburgh’, in J. Greenlees and L. Bryder, eds, *Western Maternity and Medicine, 1880–1990* (London: Pickering and Chatto, 2013), 65–80; Lindsey Earner-Byrne, ‘Twixt God and Geography: The Development of Maternity Services in Twentieth-Century Ireland’, in Greenlees and Bryder, eds, *Western Maternity,* 99–112.

9 Oakley, *Captured Womb;* Tew, *Safer Childbirth?;* Davis, *Modern Motherhood;* Sally Macintyre, ‘Consumer Reaction to Present-Day Antenatal Services’, in L. Zander and G. Chamberlain, eds, *Pregnancy Care for the 1980s* (London: Royal Society of Medicine and the Macmillan Press, Ltd., 1984), 9–10; Alex Mold, ‘Making the Patient-Consumer in Margaret Thatcher’s Britain’, *The Historical Journal,* 2011, *54,* 509–28; A. Susan Williams, *Women & Childbirth in the Twentieth Century* (Stroud: Sutton Publishing, 1997).

10 Linda Bryder, ‘What Women Want’: Childbirth Services and Women’s Activism in New Zealand, 1900–1960’, in Greenlees and Bryder, eds, *Western Maternity,* 81–98.

11 M. Hall, S. Macintyre and M. Porter, *Antenatal Care Assessed: A Case Study of an Innovation in Aberdeen* (Aberdeen: Aberdeen University Press, 1985), 109.

12
http://simd.scot/2016/#/simd2016/BTTTFTT/11/-4.0780/55.9404/ accessed 4 March, 2019.

13 This project received University ethical approval.

14 Kate Fisher found the same true in her study of British sexual behaviour. Kate Fisher, *Birth Control, Sex and Marriage in Britain, 1918–1960* (Oxford: Oxford University Press, 2006), 13. A collection of letters from English and Welsh mothers in poverty at the end of the twentieth century provides a successor to Margaret Llewelyn Davies, *Maternity: Letters from Working Women* (London: Virago, 1915; rpt. 1978) but excludes Scottish narratives. C. Gowdridge, A. Susan Williams and M. Wynn, *Mother Courage: Letters from Mothers in Poverty at the End of the Century* (London: Penguin, 1997).

15 D. M. Wegner, T. Giuliano, and P. Hertel, ‘Cognitive Interdependence in Close Relationships’, in W. J. Ickes, ed, *Compatible and Incompatible Relationships* (New York: Springer-Verlag, 1985), 253–76.

16 Maurice Halbwach’s concept of collective memory, as discussed in Barbara Misztal, *Theories of Social Remembering* (Maidenhead: Open University Press, 2003), 51.

17 Michael G. Kenny, ‘A Place for Memory: The Interface between Individual and Collective History’, *Comparative Studies in Society and History,* 1999, *41*, 420–37, 421.

18 Marie-Françoise Chanfrault-Duchet, ‘Narrative Structures, Social Models and Symbolic Representation in the Life Story’, in Sherna Berger Gluck and Daphne Patai, eds, *Women’s Words: The Feminist Practice of Oral History* (New York and London: Routledge, Chapman and Hall, 1991), 77–92, 89.

19 Chris Nottingham, ‘The Politics of Health in Scotland after Devolution’, in Chris Nottingham, ed, *The NHS in Scotland: The Legacy of the Past and the Prospect of the Future* (Aldershot: Ashgate, 2000), 173–90, 175.

20 John Stewart, *Taking Stock: Scottish Social Welfare after Devolution* (Bristol: Policy Press, 2004), 108.

21 The Scottish Office, Department of Health, *Designed to Care: Renewing the National Health Service in Scotland, Executive Summary* (Scottish Office, 1997), 5, 6.

22
*Ibid,* 11.

23 Stewart, *Taking Stock,* 58.

24 Registrar General for Scotland, Annual Report 2000; https://data.oecd.org/healthstat/infant-mortality-rates.htm. Accessed 19/05/2022.

25 C. Macdonald, *Whaur Extremes Meet: Scotland’s Twentieth Century* (Edinburgh: John Donald, 2009), 150; G. McLaren and M. Bain, *Deprivation and Health in Scotland: Insights from NHS Data* (Edinburgh, ISD Publications, 1998), ix.

26 This is not to imply a direct correlation between maternal age, low-income and single mothers.

27 National Council for the Unmarried Mother and Her Child, Annual Report 1985, 16; as cited in Thane and Evans, *Sinners? Scroungers? Saints?,* 170.

28 Vera Carstairs and Russell Morris, *Deprivation and Health in Scotland* (Aberdeen: Aberdeen University Press, 1991), 144–46.

29 Janet Greenlees, ‘”They didnae tell you nothin”’: The Failings of Sex Education, Antenatal Care, and Welfare Bureaucracies in Glasgow, *c*. 1970s–2000s', *Twentieth Century British History,* 2022, *33,* 4, 547–70, 559–60; Macdonald, *Whaur Extremes Meet,* 166.

30 P. Townsend and N. Davidson, eds, *Inequalities in Health: The Black Report and The Health Divide* (London: Penguin Book, 1980), 2, hereafter, *Black Report*. For relevant 1990s statistics, see M. Bartley and D. Blane, ‘Inequality and Social Class’, in G. Scrambler, ed, *Sociology as Applied to Medicine* (Amsterdam: Elseveir Ltd., 2008). Summary, https://www.healthknowledge.org.uk/public-health-textbook/medical-sociology-policy-economics/4c-equality-equity-policy/inequalities-distribution (accessed 9 July 2020).

31 Data from Scottish Information Services Division as discussed in J. Brotherston, ‘Inequality: Is it Inevitable?’, in C. O. Carter and J. Peel, eds, *Equalities and Inequalities in Health* (London: Academic Press, 1976), 74; *Black Report*, 74.

32 J. K. Davies, ed, *Health Promotion in Areas of Multiple Deprivation: Report of a Symposium held by the RCPSG* (Edinburgh: SHEG, 1987).

33 Margaret Whitehead, *The Health Divide,* in *Inequalities in Health* (London: Penguin, 1988), 341 and 342.

34 Davies, ed, *Health Promotion,* 60.

35
*Ibid.*

36
*Second Report from the Social Services Committee Perinatal and Neonatal Mortality*, House of Commons Paper 663-1, Session 1979–80 (London: HMSO, 1980) (Chairman: Renee Short). Hereafter, *Short Report*.

37 Whitehead, *Health Divide.*

38
*Black Report*, 143.

39
*Ibid.*

40 Whitehead, *Health Divide,* 319.

41 Hall, et al., *Antenatal Care Assessed,* 11–12.

42 There was no formal evaluation of the project. I. H. McKee, ‘Community Antenatal Care: The Sighthill Community Antenatal Scheme’, in L. I. Zander and G. Chamberlain, eds, *Pregnancy Care in the 1980s* (London: Royal Society of Medicine/Mcmillan Press, 1984), 32–41; I. H. McKee, ‘A New Look at Antenatal Care’, *World Medicine,* 1977, *12*, 72–79.

43 M. E. Reid, G. M. Gutteridge and G. M. McIlwaine, *A Comparison of the Delivery of Antenatal Care between Hospital and a Peripheral Clinic, Report of the Findings submitted to the Health Services Research Committee* (Edinburgh: Scottish Home and Health Department, April, 1983).

44 Iain Chalmers, ‘Innovation in Antenatal Care – Theory and Practice’, *Journal of the Royal Society of Medicine,* April 1984, *77*, 340–42; Macintyre, ‘Consumer Reaction’, 12.

45
*Black Report,* 2.

46 Thane and Evans, *Sinners? Scroungers? Saints?*, 169–70, 194; A. B. Atkinson, ‘Distribution of Income and Wealth’, in A. H. Halsey and J. Webb, eds, *Twentieth Century British Social Trends* (London: Macmillan, 2000), 348–94.

47 Lewis Ritchie, ‘Developing Primary Care in Scotland’, in K. Woods and D. Carter, eds, *Scotland’s Health and Health Services* (London: Nuffield, 2003), 175–215.

48
*Ibid*.

49
*Short Report*, op. cit. 86. *Reaching the Consumer in the Ante-natal and Child Health Services, Report of a Conference*, 4 April 1978 (London, DHSS, 1978; joint publication with Child Poverty Action Group). See also Jill Russell, ‘Perinatal Mortality: The Current Debate’, *Sociology of Health and Illness,* 1982, *4*, 3, 302–19, 306.

50 D. Acheson, *Independent Inquiry into Inequalities in Health: Report* (London: HMSO, 1998), hereafter *Acheson Report*, 19; *Short Report*.

51 Emphasis in original. Lindsay Reid, *Midwifery in Scotland: A History* (Erskine: Scottish History Press, 2011), 87–91, quote 89.

52 Mold, ‘Making the Patient-Consumer’.

53 Macintyre, ‘Consumer Reaction’, 9–10.

54 Hall, et al., *Antenatal Care Assessed,* 90–93.

55 Priesthill Interview, 28 August 2017.

56 Pollock Interview, 6 Nov. 2017.

57 Maryhill Interview, 22 March, 2017.

58 Chalmers, ‘Innovation’, 340.

59 Questionnaires and Maryhill Interviews, 22 March and 26 July, 2017; Pollock Interview, 6 November 2017.

60 Questionnaires from all focus groups.

61 Maryhill Interview, 22 March 2017; Priesthill Interview, 28 August 2017.

62 Mold, ‘Making the Patient-Consumer’.

63 Maryhill Interviews, 22 March and 26 July, 2017; Priesthill Interview, 28 August 2017; Pollock Interview, 6 November 2017.

64 Maryhill Interview, 26 July 2017.

65
*Ibid.*

66 Priesthill Interview, 28 August 2017.

67 Angela Davis, ‘Women’s Experiences of the Maternity Services in Berkshire and Oxfordshire, *c.* 1970–1990’, in Greenlees and Bryder, eds, *Western Maternity,* 129–46; Davis, *Modern Motherhood,* 84–101.

68 Davis, ‘Women’s Experiences’; Davis, *Modern Motherhood,* 97.

69 Priesthill Interview, 28 August 2017.

70 Jacqueline Wolf, *Caesarean Section: An American History of Risk, Technology and Consequence* (Baltimore: Johns Hopkins University Press, 2018).

71 Davis, ’Women’s Experiences’; Tew, *Safer Childbirth?*, 139–40.

72
*Ibid.*

73 Davis, ‘Women’s Experiences’; Davis, *Modern Motherhood,* 84–101.

74 Davis, ‘Women’s Experiences’, 146, 140–43.

75 Greenlees, ‘“They didnae tell you nothin”.

76 Maryhill Interviews, 22 March and 26 July, 2017; Priesthill Interview, 28 Aug. 2017; and Pollock Interview, 6 Nov. 2017. Quote from Maryhill Interview, 22 March 2017.

77 Priesthill Interview, 28 August 2017.

78 Hall, Macintyre and Porter explain these issues in relation to Aberdeen. Hall, et. al., *Antenatal Care Assessed*, 79–82.

79 Low-income LatinX patients have also been found to appreciate prenatal care providers who seemed ‘worried’ about them. See Lauren Trainor, Ellen Frickberg-Middleton, Monica McLemore, Linda Franck, ‘Mexican-Born Women’s Experiences of Perinatal Care in the United States’, *Journal of Patient Experience,* 2020, 1–5.

80
*Ibid.*

81 One Parent Families Archive, National Council for the Unmarried Mother and Child, undated, Glasgow Caledonian University Archives; http://Historic-hospitals.com/gazetteer/Glasgow. Accessed 14 June, 2022.

82 Maryhill Interview, 26 July, 2017; Pollock Interview, 6 Nov. 2017.

83 Pollock Interview, 6 November 2017. The Govan branch of the Innocents and Saint Francis’ Maternity Hospital have closed.

84 Pollock Interview, 6 November 2017.

85 LR Interviews 116, in Reid, *Midwifery in Scotland,* 90–91.

86 Reid, *Midwifery,* 153.

87 Pollock Interview, 6 November 2017.

88 Priesthill Interview, 28 August 2017; Pollock Interview, 6 November 2017; Maryhill Interviews, 22 March and 26 July 2017.

89
*Ibid.*

90 Davis, ‘Women’s Experiences’.

91 Pollock Interview, 6 November 2017.

92 Maryhill Interview, 26 July 2017.

93 Maryhill Interview, 22 March 2017.

94
*Ibid.* Maryhill interviews, 22 March and 26 July 2017; Priesthill Interview, 28 August 2017.

95 Maryill Interview, 26 July 2017.

96 Maryhill Interview, 22 March 2017.

97 Maryhill Interviews, 22 March and 26 July 2017; Priesthill Interview, 28 August 2017; Pollock Interview, 6 November 2017.

98 Davis, *Modern Motherhood, 154.*

99 Maryhill Interview, 22 March 2017.

100
*Ibid.*

101
*Ibid.*

102 Roger Davidson and Gayle Davis, *The Sexual State: Sexuality and Scottish Governance, 1950–80* (Edinburgh: Edinburgh University Press, 2012).

103 For more on the lengthy influence of religious morality in Scottish healthcare, see Roger Davidson, *Dangerous Liaisons: A Social History of Venereal Disease in Twentieth-Century Scotland* (Amsterdam: Rodopi, 2000), 327; Davison and Davis, *Sexual State*; Janet Greenlees, ‘The Peculiar and Complex Female Problem’: the Church of Scotland and health Care for Unwed Mothers, 1900–1948’, in Greenlees and Bryder, *Western Maternity,* 47–64; Thane and Evans argue that late twentieth-century English politics and society reverted to blaming the victim, the unwed mother, but without the religious overtones of earlier. *Sinners? Scroungers? Saints?,* 169–208.

104 K. Hinds and L. Jamieson, ‘Rejecting Traditional Family Building? Attitudes to Cohabitation and Teenage Pregnancy in Scotland’, in J. Curtice, D. McCrone, A. Park and L. Paterson, eds, *New Scotland, New Society?: Are Social and Political Ties Fragmenting?* (Edinburgh, Edinburgh University Press: 2002), 37, 38, 40.

105 Davidson and Davis, *Sexual State,* 295–96.

106
*Ibid.,* 299.

107 Hall, et. al, *Antenatal Care Assessed,* 6–8.

108 Maryhill Interview, 22 March 2017; Pollock Interview, 6 November 2017.

109 Pollock Interview, 6 November 2017.

110
*Ibid*.

111
*Ibid*.

112
*Ibid*. Lara Freidenfelds has argued for better terms or metaphors surrounding miscarriage. While ‘embryos’ or ‘foetuses’ or ‘baby’ are the current technical terms, the term ‘sprout’ might make more acceptable the possibility that a pregnancy does not continue to completion. L. Freidenfelds, *The Myth of the Perfect Pregnancy: A History of Miscarriage in America* (New York: OUP, 2020).

113 Pollock Interview, 6 November 2017; Maryhill Interview, 26 July 2017.

114 Priesthill Interview, 28 August 2017.

115
*Ibid.*

116 Reid, *Midwifery in Scotland,* 139–64.

117 Oral History LR 85, as cited in *Ibid,* 139.

118
*Ibid,* 155–57.

119
*General Register Office for Scotland.*

120 Janet Greenlees, ‘To Care and Educate: The Continuity Within Queen’s Nursing in Scotland, c. 1948–2000, *Nursing History Review,**26*, 1, 2018, 97–110.

121 Reid, *Midwifery in Scotland,* 160.

122 Tania McIntosh, *A Social History of Maternity and Childbirth: Key Themes in Maternity Care* (London and New York: Routledge, 2012), 135.

123 Pollock Interview, 6 November, 2017; Maryhill Interviews, 22 March and 26 July, 2017; Priesthill Interview, 28 August 2017.

124 Priesthill Interview, 28 August 2017.

125 Williams, *Women and Childbirth*, 257–58.

126 Thane and Evans, *Sinners? Scroungers? Saints?,* 169.

127 L. Jamieson, ‘Changing Intimacy: Seeking and Forming Couple Relationship’, in Lynn Abrams and Callum Brown, eds, *A History of Everyday Life in Twentieth-Century Scotland* (Edinburgh: Edinburgh University Press, 2010), 76–102, 84.

128
*Black Report,* 143.

129 Maryhill Interview, 22 March 2017.

130 Hall, et. al., *Antenatal Care Assessed;* McKee, ‘Community Antenatal Care’; McKee, ‘New Look’; Chalmers, ‘Innovation’.

131 Andrew Tannehill, ‘Promoting Health in Scotland’, in K. Woods and D. Carter, eds, *Scotland’s Health and Health Service* (London: The Stationary Office, 2003).

132 For example, Judith Houck, *Hot and Bothered: Women, Medicine, and Menopause in Modern America* (Cambridge, MA: Harvard University Press, 2006); Linda Bryder, *Women’s Bodies and Medical Science: An Inquiry into Cervical Cancer* (Houndsmills: Palgrave Macmillan, 2010); Bryder, ‘What Women Want’; and Davis, *Modern Motherhood,* 107.

## Notes

Janet Greenlees is a Reader in Health History at Glasgow Caledonian University. She has published widely on American and British maternal health and healthcare, occupational, and environmental health. Her most recent book, *When the Air became Important: A Social History of the New England and Lancashire Textile Industries* was published by Rutgers University Press in 2019. Her current research investigates relationships between poverty, vulnerability and maternity care.

